# Psychological Stress and 30-Day All-Cause Hospital Readmission in Acute Coronary Syndrome Patients: An Observational Cohort Study

**DOI:** 10.1371/journal.pone.0091477

**Published:** 2014-03-12

**Authors:** Donald Edmondson, Philip Green, Siqin Ye, Hadi J. Halazun, Karina W. Davidson

**Affiliations:** Center for Behavioral Cardiovascular Health, Department of Medicine, Columbia University Medical Center, New York, New York, United States of America; University of Utah, United States of America

## Abstract

**Background:**

Many acute coronary syndrome (ACS; myocardial infarction and unstable angina) patients are rehospitalized within 30 days of discharge, and recent US health policy initiatives have tied hospital Medicare reimbursement to 30-day readmission rates. Patient-perceived psychological stress is thought to impact prognosis after ACS. A recently offered “posthospital syndrome” model of 30-day readmissions posits that the stress level at the time of the index hospitalization itself may increase 30-day risk for readmission in ACS patients. We tested whether self-reported stress in the days surrounding the ACS hospitalization was associated with increased risk for readmission within 30 days.

**Methods:**

A mean of 8.5 days after discharge, 342 consecutively hospitalized ACS patients reported on how often they felt stress during the past two weeks. Readmission within 30 days of hospital discharge for any cause was determined by follow-up telephone calls to patients and confirmed by hospital records.

**Results:**

Overall, 40 (11.7%) participants were readmitted within 30 days, and 22 (6.4%) reported high stress. Readmission within 30 days was more common in patients with high stress (5 admissions, 23%) than in patients with low stress (35 admissions, 11%). After adjustment for demographic and clinical factors, as well as depression, high stress was associated with a 3-fold increased risk of 30-day readmission (HR = 3.21, 95% CI = 1.13, 9.10).

**Conclusions:**

Previous research has shown that stress in the days surrounding a hospitalization can mark long-term cardiovascular risk, but this is the first study to test a hypothesis of the posthospital syndrome model of early readmission. Further research is needed to confirm the association between stress and readmission risk, and to identify the processes of hospitalization that could be modified to both reduce the stress experienced and that would also be effective for reducing readmissions.

## Introduction

Within the first 30 days of discharge from the hospital for an acute myocardial infarction (MI), 1 out of every 5 patients aged 65 years and older are readmitted [1]. This high proportion of readmissions has led federal policy makers to include a provision in the Affordable Care Act that reduces Medicare payments to hospitals based on their 30 day readmission rate. In an issue of *JAMA* devoted to readmissions, one study of over 500,000 patients hospitalized for MI found that more than 100,000 were readmitted within 30 days [1]. Surprisingly, only 10% of these readmissions were for recurrent MI, and only half were for any cardiovascular reason. Thus, patients recently discharged for acute coronary syndrome (ACS; MI and unstable angina) are at high risk for 30-day readmissions, very often for causes unrelated to ACS.

Hospitalization for ACS is a highly stressful experience [2], in particular due to the unfamiliarity of the surroundings, loss of control, fear, and lack of information that patients often endure. Some evidence suggests that the stress level during hospitalization is particularly related to long-term risk for adverse cardiac outcomes in ACS patients. [3] A meta-analytic review of 43 studies showed that stress reduction interventions in these patients can reduce two-year mortality in men and event recurrence in all CHD patients by 27%. [4] No trials have yet examined the effects of stress reduction on early readmission after an ACS event.

One particularly potent cause of perceived stress level at the time of any hospitalization may be hospitalization itself. Krumholz has offered a model in which he suggests that, at hospital discharge, patients are not only recovering from their initial illness but are also recovering from the additional burden of a very psychologically and physiologically stressful hospitalization experience [5]. Because of this stress endured in the days leading up to the hospitalization, in-hospital, and then in the days immediately post-discharge, patients' “physiological systems are impaired, reserves are depleted, and the body cannot effectively defend against health threats.” Therefore, regardless of the reason for the initial hospitalization, perceived stress levels during the days surrounding the hospitalization cause a short-term window of heightened generalized vulnerability for readmission— termed Post-Hospital Syndrome (PHS). Although many interventions have been tested to reduce iatrogenic aspects of the hospitalization, readmission rates remain high [6].

The PHS model hypothesizes that risk markers for 30-day readmission are manifested by reports of perceived stress, and possible sources of these increases in reports of stress could include sleep deprivation, physical deconditioning, and weight loss. It is possible that stress levels during the hospitalization are implicated in the poor prognosis and increased 30-day readmission of patients, but the association of perceived stress level and 30-day readmission as not yet been documented. We tested the association of peri-hospitalization stress to 30-day readmission by examining whether acute coronary syndrome (ACS; MI, unstable angina) patients who report high levels of stress in the two weeks surrounding the hospitalization are at increased risk for 30-day readmission.

## Methods

This study received ethics approval by the Institutional Review Board (IRB) of Columbia University Medical Center (# IRB - AAA9286) and was conducted in accordance with the Declaration of Helsinki. All study patients were recruited from the clinical departments at Columbia University. Written informed consent was obtained from all study patients. Completed informed consent documents were then stored in a secure location as per Columbia University IRB protocol.

Patients hospitalized for ACS were recruited from Columbia University Medical Center within one week of admission as part of the Prescription Use, Lifestyle, Stress Evaluation (PULSE) study. The PULSE study is an observational, single-site, prospective cohort study of the prognostic risk conferred by depressive symptoms and clinical depressive disorders at the time of an acute coronary syndrome (ACS). Patients reported demographic and psychosocial data during hospitalization, and reported on their stress level during and after their hospitalization by telephone interview after hospital discharge (mean 8.5 days), using a single item, “During the past two weeks, how often have you felt tense or ‘wound up.’” Responses were dichotomized (“often” or “most of the time” = 1; “rarely” or “never” = 0). Rehospitalization within 30 days of hospital discharge for any cause was determined by follow-up telephone calls to patients and confirmed by hospital records. Additionally, hospital records were proactively searched for rehospitalizations.

We conducted a Cox regression analysis with adjustment for demographics, ACS type (myocardial infarction vs unstable angina), the Global Registry of Acute Coronary Events (GRACE) risk score [7], Charlson comorbidity index [8], baseline left ventricular ejection fraction (LVEF)<40, and depression symptom score as measured with the Beck Depression Inventory (BDI)≥10 [9]. Participants rehospitalized before stress assessment were removed from analyses.

## Results

**Figure 1:**
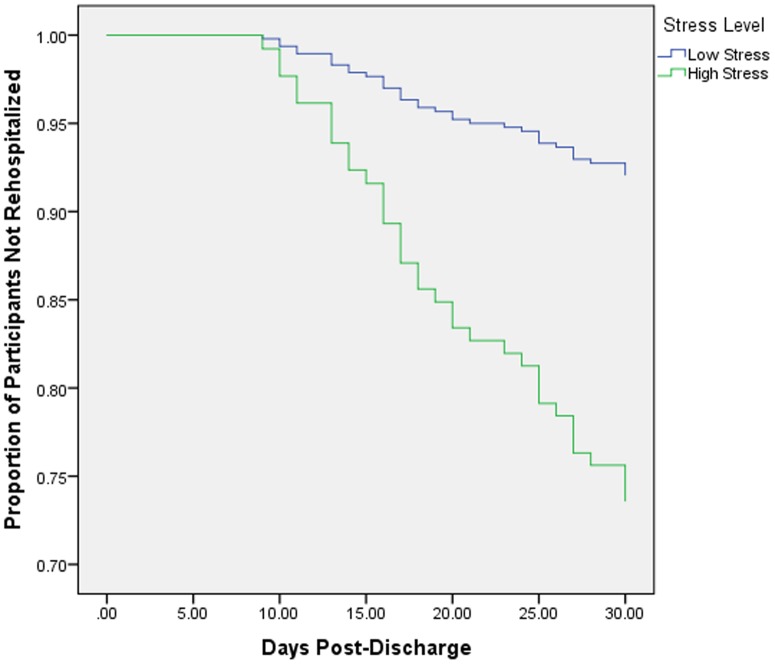
Cox proportional hazards regression analysis predicted survival curves for acute coronary syndrome (ACS) patients by stress category, adjusted for age, sex, race, ethnicity, type of ACS, Charlson comorbidity index score, Global Registry of Acute Coronary Events risk score, and left ventricular ejection fraction.

Of 342 participants (**Table 1**), 40 (11.7%) were rehospitalized within 30 days, and 22 (6.4%) reported high stress. Rehospitalization within 30 days was more common in patients with high stress (5 events, 23%) than in patients with low stress (35 events, 11%). High stress patients had significantly more comorbidities than low stress (2.59 vs. 1.55, p<.01) and were more likely to be depressed (15% vs. 2%, p<.001), but did not differ on any other covariate. High stress during index hospitalization was associated with increased risk for early rehospitalization in both unadjusted (HR = 2.29, 95% CI = 0.90, 5.86) and fully adjusted models (HR = 3.21, 95% CI = 1.13, 9.10; **Figure 1**).

**Table 1:** Participant characteristics by stress category.

Characteristic	Full sample (N = 342)	High Stress (N = 22)	Low Stress (N = 320)
Age, y	62.4±11.6	61.2±9.9	62.3±11.6
Male, *n* (%)	225 (66)	11 (50)	214 (67)
Black/African American, *n* (%)	82 (24)	8 (36)	74 (23)
Hispanic, *n* (%)*	139 (41)	8 (36)	131 (41)
MI as index ACS, *n* (%)	177 (52)	7 (32)	170 (53)
GRACE risk score	91.6±29.2	88.1±26.3	91.6±29.6
LVEF≤40, *n* (%)	51 (15)	3 (14)	48 (15)
Charlson comorbidity index	1.6±1.7	2.6±2.0	1.6±1.7
Beck Depression Inventory >10***	124 (36)	18 (82)	106 (33)

Note: * *p*<.05, *** *p*<.001. Abbreviations: GRACE, Global Registry of Acute Coronary Events; LVEF, Left ventricular ejection fraction; MI, myocardial infarction;

Note: Values are mean ± SD unless indicated otherwise.

To explore whether the stress that participants reported was associated in part with the hospitalization itself, we calculated the correlation between the stress item and self-reported intrusive thoughts about the ACS hospitalization 1 month later using the intrusions subscale of the Impact of Events Scale-Revised (IES-R), specifically keyed to the ACS hospitalization [10]. The correlation between the stress item and intrusive thoughts about the ACS hospitalization was .20, p<.001.

## Discussion

Perceived stress is a known risk factor for incident cardiovascular events [11], and anxiety and depression immediately before and after cardiovascular hospitalization are associated with 6-month readmission risk [12], [13]. Although previous studies have shown that stress is associated with poor long-term prognosis in ACS patients [3], [14], to our knowledge, this is the first study to test the association between 30-day readmission risk and perceived stress in the days surrounding the index hospitalization. We found that patients who report feeling high stress during the time of an index hospitalization are at significantly greater risk for 30-day rehospitalization than their less stressed counterparts. Interestingly, neither GRACE risk score nor Charlson comorbidity index were significant predictors of 30-day readmission.

The mechanisms linking perceived stress to adverse cardiovascular outcomes and readmission are likely many, including increased activity of the hypothalamic pituitary axis [15], autonomic imbalance [16], behavioral factors, platelet activation and/or endothelial dysfunction [17], all of which would contribute to increased risk of an adverse cardiovascular event or an early readmission.

In part due to our measurement strategy, there may be a number of explanations for our finding that self-reported stress is associated with readmission risk. For example, subjective stress during hospitalization could occur as the result of disturbed sleep, physical deconditioning, and/or weight loss both during hospitalization and in the days after discharge, and therefore serve as a marker for the depletion of physiologic reserve associated with readmission. Conversely, subjective stress during hospitalization may increase inflammation and risk for arrhythmia and cognitive dysfunction, leading to adverse outcomes such as readmissions.

Our one-item measure of self-reported stress over a two-week time period may have captured stress due to a number of factors other than the hospitalization, such as socioeconomic status, work stress, or family stress. To partially explore this possibility, we examined the association of the 1-item stress measure with subsequent intrusive thoughts specific to the ACS hospitalization, and found that the two were positively correlated—though the association was small. High perceived psychological stress is associated with both incident[11] and recurrent cardiac events.[3] For example, a recent study found that high financial stress after ACS was associated with increased risk for readmission at 1 year [18]. Thus, further research is needed to better characterize the different sources of stress contributing to overall perceived stress in the hospitalization time period, and so more precisely determine which aspects of subjective stress most warrant intervention trials to determine if they in turn reduce readmission risk.

There are also several limitations to our study. As a retrospective analysis of a small sample, our findings are necessarily hypothesis generating. Given our modest sample size, it is also possible that our findings may be influenced by chance. Nonetheless, our findings are consistent with the robust literature on the adverse health outcomes associated with perceived stress. As we detected a significant relationship between perceived stress and readmission risk despite our small sample size, inadequate power was not an issue. As discussed above, our study is also limited by the use of retrospective report to determine participants' stress in the past two weeks, which included the hospitalization and a few days prior and after the actual hospitalization. Finally, we used a single item to assess stress, and single-item measures tend to be less reliable than multiple-item assessments of a construct. However, low reliability in scales tends to lead to an attenuation of observed associations between a single-item scale and an outcome, suggesting that we may have seen a stronger association of stress and rehospitalization risk had we used a multiple item assessment of perceived stress. Despite these limitations, this is the first study to attempt to test one hypothesis of the post-hospital syndrome model; that perceived stress during the time of a hospitalization may in part be responsible for some of the high readmission rates occurring in many of our hospitals around the country. As such, our first study in this area offers intriguing support for efforts to reduce readmission rates through improvements to the hospitalization experience that may reduce the stress burden experienced by our patients.

## Conclusions

Future studies should be conducted with larger samples sizes and with stress measures that more precisely isolate the sources of stress responsible for excess readmission in post-ACS patients. Should we discover that the sources of stress responsible for early readmission are modifiable processes of the hospitalization, then improving upon these processes may have the dual benefit of reducing patient stress during hospitalization while also reducing early readmission in ACS patients.
